# Phenolic and Nonpolar Fractions of *Elaeagnus rhamnoides* (L.) A. Nelson Extracts as Virulence Modulators—In Vitro Study on Bacteria, Fungi, and Epithelial Cells

**DOI:** 10.3390/molecules23071498

**Published:** 2018-06-21

**Authors:** Barbara Różalska, Beata Sadowska, Jerzy Żuchowski, Marzena Więckowska-Szakiel, Aleksandra Budzyńska, Urszula Wójcik, Anna Stochmal

**Affiliations:** 1 Department of Immunology and Infectious Biology, Institute of Microbiology, Biotechnology and Immunology, Faculty of Biology and Environmental Protection, University of Lodz, Banacha 12/16, 91-237 Lodz, Poland; beata.sadowska@biol.uni.lodz.pl (B.S.); marzena.wieckowska@biol.uni.lodz.pl (M.W.-S.); urszula.wojcik@poczta.fm (U.W.); 2 Department of Biochemistry, Institute of Soil Science and Plant Cultivation, State Research Institute, Czartoryskich 8, 24-100 Puławy, Poland; jzuchowski@iung.pulawy.pl (J.Ż.); asf@iung.pulawy.pl (A.S.); 3 Laboratory of Microbiological and Technical Services, Institute of Microbiology, Biotechnology and Immunology, Faculty of Biology and Environmental Protection, University of Lodz, Banacha 12/16, 91-237 Lodz, Poland; aleksandra.budzynska@biol.uni.lodz.pl

**Keywords:** *Elaeagnus rhamnoides* butanol extracts, phenolic, nonpolar fractions, antivirulence properties

## Abstract

Butanol extracts from leaves, twigs, and fruits of *Elaeagnus rhamnoides* (L.) A. Nelson (sea buckthorn, SBT) were fractionated into phenolic and nonpolar lipid components, the chemical composition of which was analyzed. Assuming that an effect on natural microbiota and host epithelial cells needs to be assessed, regardless of the purpose of using SBT formulations in vivo, the minimal inhibitory/biocidal/fungicidal concentrations (MICs/MBCs/MFCs) of the fractions and reference phytocompounds were screened, involving 17 species of Gram-positive and Gram-negative bacteria and *Candida* species. The MICs of SBT extracts were in the range of 0.25–2.0 mg∙mL^−1^. Since direct antimicrobial activity of the extracts was quite low and variable, the impact of subMIC on the important in vivo persistence properties of model microorganisms *S. aureus* and C. *albicans* was evaluated. Tests for adhesion and biofilm formation on an abiotic surface and on surfaces conditioned with fibrinogen, collagen, plasma, or artificial saliva showed the inhibitory activity of the fractions. The effects on fluorescein isothiocyanate (FITC)-labeled staphylococci adhesion to fibroblasts (HFF-1) and epithelial cells (Caco-2), and on fungal morphogenesis, indicated that SBT extracts have high antivirulence potential. Cytotoxicity tests (MTT reduction) on the standard fibroblast cell line showed variable biological safety of the fractions depending on their composition and concentration. The new information afforded by this study, additional to that already known, is of potential practical value in the application of SBT-derived preparations as antivirulence agents.

## 1. Introduction

The ongoing therapeutic crisis connected with antibiotic resistance has led to the launch of an intensive search for alternative ways of fighting infections. Plant-derived extracts/compounds intended for individual use or in combination with classical drugs are seriously being considered and tested worldwide. The main advantage is usually in their equal effectiveness, irrespective of the particular drug susceptibility profile of a microorganism and its preferred growth phenotype (sessile or planktonic). No less important is the fact that, due to the complex composition of the plant extracts and different mechanism of single phytocompound action, the risk of microbial resistance developing is relatively low. These positive properties have attracted even more attention over the years, with questions being asked as to what can be offered instead of chemotherapeutics that are ineffective in combating, for example, difficult-to-eradicate biofilm-related infections. This is because there is a widely accepted opinion, supported by clinical observation, that the majority of bacteria and microscopic fungi form highly drug-tolerant/resistant free-floating aggregates and/or biofilms attached to abiotic surfaces (medical devices), or can live on the necrotic surface of host tissues [1,2,3,4,5].

The aim of the study was to assess the antimicrobial/antibiofilm activity of butanol extracts from the leaves, twigs, and fruits of *Elaeagnus rhamnoides* (L.) A. Nelson (sea buckthorn, SBT) fractionated into phenolic and nonpolar components to find those that are the most active. The main goal was to check whether phenotypic features of *Staphyloccoccus aureus* bacteria and *Candida albicans* yeasts, which determine their success as invasive pathogens, could be the possible targets. A number of products of *E. rhamnoides* (previously known as *Hippophae rhamnoides*) have been used for centuries as dietary supplements and also in folk medicine against a wide range of diseases. Currently, they are being applied in some divisions of modern medical practice and the cosmetic industry. Due to the lack of standardization of plant phytocompounds, there is usually no common or scientific belief about the pro-health situation that can fully justify the use of SBT-derived products. Indeed, it seems that extracts from different parts of this plant show diverse and interesting activities in vitro, including antioxidant, anti-inflammatory, antithrombotic, anticancer, antimicrobial, and many other properties. However, the available data concerns mainly sea buckthorn fruit-derived products, although other parts of this plant have also proven to be efficient sources of biologically active ingredients [6,7,8,9]. In our previously published study, we provided some original evidence that SBT-derived extracts of leaves, and even of waste twigs, possessed significant activity affecting important *Candida* spp. virulence factors and showing synergism with antimycotic drugs [6]. Nonetheless, there is little interest in the wider use of the antimicrobial potential of SBT compounds in medical practice, although there is plenty of evidence of their direct biocidal effect in vitro, ex vivo, and in animal models. From the above (microbiological) range, no ongoing or planned clinical study has been found on the ClinicalTrials.gov portal [10]. In 2018, this website showed that only 5 of 12 registered studies on SBT have been completed, but data on them are still unavailable. The phenolic fraction from fruit extract was used in only one trial. In the other cases, unfractionated extracts—mainly from fruits (1 from leaves) or intact SBT oil—were tested. In general, the goal was to demonstrate the beneficial effects of SBT-containing dietary supplements in patients with type 2 diabetes, notably in reducing obesity, improving eye health, and relieving inflammation of the mucous membranes. New clinical trials, on which there is little information of their status or implementation, refer to the topical usage of SBT oil in cream formulations for dermatology and gynecology. The latest trial to be announced, with a pronounced immunological inclination, concerns the impact of a single dose of SBT berry-based proanthocyanidin extract on adult stem cells. A number of different types of stem cells will be tested to examine the effect on cell mobilization and homing after being treated with plant-based extracts. Therefore, there is much to be done regarding the potential use of sea buckthorn products as antimicrobials. The objects of our study belong to the group of opportunistic bacterial/fungal pathogens well equipped with pathogenicity factors. At individual stages of infection, they are successively and successfully expressed, depending on the changing conditions of the host microenvironment, which makes these microorganisms “difficult opponents”.

## 2. Results and Discussion

One of the main questions asked herein was which of the sea buckthorn (SBT)-derived phenolic and nonpolar fractions finally separated from butanol extracts has better antimicrobial activity. Details of fraction preparation and component characterization are shown in Supplementary Materials. Briefly, chemical analysis of fractionated SBT extracts from leaves, twigs, and fruits indicated significant differences in their main constituents. In the phenolic fraction of leaf extract, hydrolysable tannins/ellagotannins and triterpenoid saponins dominate; the twig-derived fraction is rich in compounds of the proanthocyanidins/catechin type; and isorhamnetin glycosides dominate in the fruit-derived preparation. The nonpolar fraction of leaf extract contains a high amount of triterpenoid saponins, which are virtually absent in the two other source fractions (below 5%), which are richest in triterpenoids, including those acylated with phenolic acids (Table 1).

The relative content of individual compound groups in the fractions of SBT leaf and twig extracts is expressed as a percentage of the total peak area (Corona Charged Aerosol Detector, CAD) and is presented in Tables S1 and S2. Secondary metabolites in these fractions, as the listed compounds corresponding to UHPLC-CAD peaks (with area ≥1% of the total peak area), are given in Tables S3–S6. Data concerning the individual composition of SBT fruit-derived fractionated extract are not presented here since they have already been published [11]. Considering the demonstrable differences in composition of the SBT fractions, one could expect them to have different antimicrobial activities, usually dependent on a quantitatively dominating group of the compounds. This assumption was supported by the results of our previous study in which the high antifungal activity of the proanthocyanidins-rich fraction of SBT twig extract was reported. It almost equaled the effectiveness of the same type of fraction separated from leaf extract, rich in hydrolysable tannins [6]. The present research was to determine whether further fractionation of the extracts results in changes or not, i.e., either increases or decreases their activity.

### 2.1. Direct Antimicrobial Activity of Fractionated SBT Extracts

Starting from the unquestionable beneficial health effects of SBT-based products as nutrients or dietary supplements, we go further in looking for justification for their use in more specialized areas of medical practice than previously proposed. The search for agents that turn off the production of virulent factors or diminish their expression can bring about a new generation of species-specific antivirulent drugs. In the case of SBT products, they have applications in the supportive therapy of local skin lesions, mucosal infections, and associated inflammatory symptoms in wound healing and other conditions [8,9,11,12,13,14,15,16,17,18], directions that are in part reflected in the topics of ongoing clinical trials [10]. However, the sites of possible action of SBT-derived compounds in human body are the niches richly inhabited by many microorganisms constituting natural microbiota, which play a significant role in the host immune system [19,20]. Therefore, the influence of fractionated SBT extracts has been tested on representatives of pathogenic, opportunistic, and commensal microorganisms of the following genera: *Staphylococcus*, *Streptococcus*, *Helicobacter*, *Bacillus*, *Escherichia*, *Proteus*, *Pseudomonas*, *Lactobacillus*, *Candida*. Individual species from these genera are present on the skin and the mucous membranes of the oral cavity, colonize the gastrointestinal tract and the urethra, and constitute part of the microbiome of the vaginal mucosa [3]. Our screening experiment showed that Minimal Inhibitory Concentrations (MICs) of the fractions varied depending on the type (phenolic or nonpolar), the origin of the extract (different organs of the plant), and the target microorganism. The nonpolar fraction separated from all vegetative parts of SBT had no direct antimicrobial activity over the concentration range that was tested, the only exception being activity against *C. albicans* ATCC 10231 at an MIC level of 1 mg·mL^−1^. The phenolic fraction of SBT fruit extract was also inactive (MICs > 1.0 mg·mL^−1^). However, such phenolic fractions obtained from the extracts of leaves and twigs expressed moderate activity to a comparable degree (Table 2). It was noted, however, that their action was stronger against Gram-positive than Gram-negative bacteria, and against most species of the yeasts, resulting from known differences in cell wall structure. Noteworthily positive is the low sensitivity of the Gram-positive “probiotic” bacteria, *Lactobacillus acidophilus*, inhabiting various ontocenoses of the human body, and of Gram-negative intestinal bacilli of the *E. coli* species. On the other hand, Gram-negative *Helicobacter pylori*, colonizing the stomach mucosa of more than half the population, as well as *Proteus vulgaris*, present in the microbiome of the gastrointestinal and urinary tracts, are characterized by having only an average degree of sensitivity to these products.

The MICs of reference compounds, adapted to their qualitative/quantitative representation in the fractions against selected bacterial and fungal strains (hereinafter explored in more detail), are presented in Table 3. Only ursolic acid used separately was substantially biostatic. However, the most frequent additive or hyperadditive synergy of the individual components occurs when combined.

The question is whether such a range of activity of fractionated SBT products is to their advantage or disadvantage. In our opinion, this is valuable positive information that can be used in the future to develop targeted “personalized” therapy. This suggestion does not differ fundamentally from the idea of the differential action of antibiotics and chemotherapeutics, as in the saying “everything does not work for everything”. Unfortunately, proposals/suggestions for scientifically justified targeted and “personalized” use of the antimicrobial potential of plants, including SBT-derived products, are not easily introduced for many reasons. Our data and those in numerous other reports refer to different kinds of extracts, and they may not always have been sufficiently well characterized. Moreover, different microbiological methods with noncomparable specificity and sensitivity are used for the abovementioned purposes. Hence, interlaboratory comparison of the results from a given range of studies on their biological activity is difficult, unreliable, and not very constructive [15,16,18].

### 2.2. Antivirulence Properties of Fractionated SBT Extracts

We have researched other possibilities of using phytochemicals than with their direct microbicidal activity. At least three clinical situations associated with infection and inflammation may be considered regarding the topical use of SBT products. Above all, chronic wound infection, other skin or oral infections with mixed etiology, and vulvovaginitis caused by bacteria and fungi can be mentioned. In all of them, the participation of the microorganisms we examined, *S. aureus* and *C. albicans*, that can form sessile (biofilm) populations, is significant. It is well known that most biofilm-associated infections are connected to a discontinuity in the skin, mucosa, and/or the underlying tissues. These are easily invaded due to normal colonization of the given portal by physiological and environmental microbiota. Moreover, damaged tissues are much more susceptible to colonization because of local oxygen deficiency, necrosis, or a lower activity of vascular endothelial cells and fibroblasts participating in repair. The most significant problem related to the treatment of biofilm infections, irrespective of microbial origin and localization, is their resistance/tolerance to antibiotics. This results not only from an increased number of drug-resistant microorganisms, but from facilitated gene transfer within the biofilm, as well as their very unique structure and physiology [21,22,23].

#### 2.2.1. Adhesion and Biofilm Formation

Few mechanisms of antibiofilm action are considered for many natural products: direct biocidal activity, inhibition of the expression of adhesins, and interruption of intercellular communication. All of the suspected mechanisms have been reflected in our study. By reproducing in vitro conditions simulating real situations in vivo that bacteria and fungi can theoretically encounter during infection, target surfaces for adhesion and biofilm formation conditioned with extracellular matrix proteins (ECM) have also been assessed, besides inert surfaces. To mimic wound beds or mucosa of the oral cavity/gastrointestinal tract, the surfaces were coated with fibrinogen, collagen, blood plasma, and artificial saliva. *S. aureus* can adhere to and invade tissues/host cells usually with the participation of the surface MSCRAMMs (microbial surface components recognizing adhesive matrix molecules) family. Similarly, *Candida* yeasts possess numerous cell surface structures that help their adhesion to the surface of medical polymers or tissues “decorated” with ECM molecules [22,23,24,25,26].

**Figure 1:**
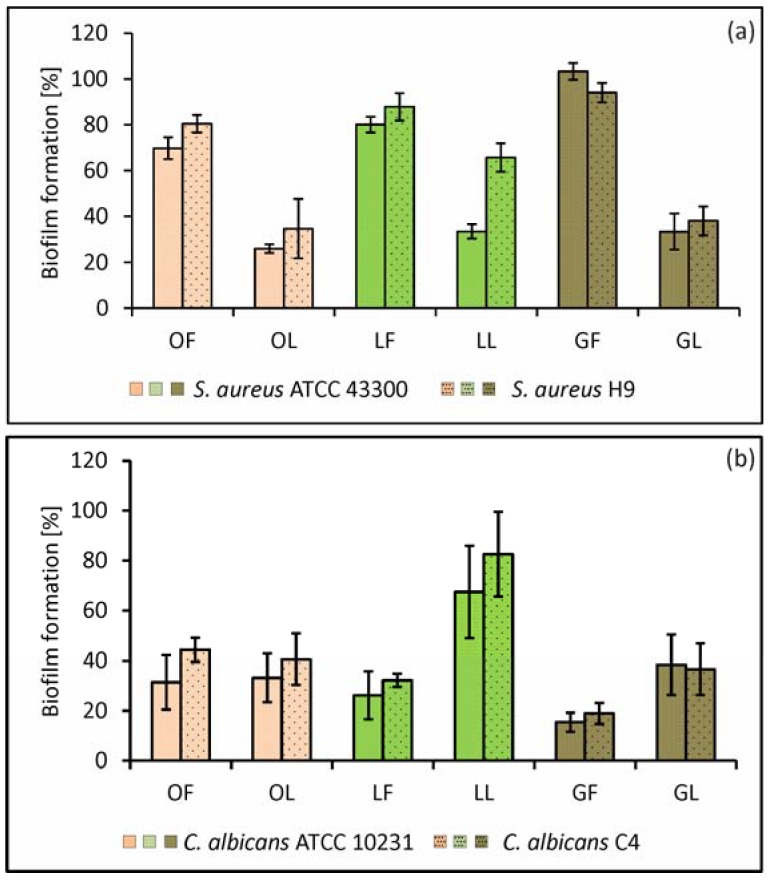
Biofilm formation of *Staphylococcus aureus* ATCC 43300 and clinical *S. aureus* H9 (**a**); *Candida albicans* ATCC 10231 and clinical *C. albicans* A4 (**b**); on the abiotic (polystyrene) surface in the presence of subMICs of fractionated SBT extracts. LF, GF, OF denote phenolic fractions of leaf, twig, and fruit-derived extracts, respectively; LL, GL, OL denote nonpolar (lipid) fractions of leaf, twig, and fruit-derived extracts, respectively. Inhibitory effect was analyzed in terms of metabolic activity of biofilm mass by LIVE/DEAD BacLight Bacterial Viability kit (*S. aureus*) and the “fluorescein diacetate (FDA) reduction” method (*C. albicans*). The percentage ± S.D. of biomass activity compared with the control (untreated), which was considered as 100%, is presented.

At this stage, we have demonstrated that both types of SBT-derived fractions have in vitro anti-adhesive properties against *S. aureus* ATCC 43300 reference and *S. aureus* H9 clinical isolate (MRSA), as well as against fungi *C. albicans* ATCC 10231 reference and *C. albicans* C4 clinical isolate (from a patient’s stool) (Figure 1). Despite the relatively weak direct biostatic/biocidal activity of SBT preparations, at 0.5× MIC (0.125, 0.25, or 0.5 mg·mL^−1^), they strongly inhibited microbial adhesion to an inert surface (up to 45.9 ± 2.7%, *p* = 0.0199 and 75.2 ± 3.7%, *p* = 0.008 for *S. aureus* and *C. albicans*, respectively). The use of subinhibitory concentrations of these products during in vitro studies is justified by the presence of similar conditions in vivo. From the pharmacodynamic/pharmacokinetic analysis, it is clear that in soft tissues (e.g., subcutaneous layers, intestine, and lung mucosa), the pathogens or physiological microbiota might only be exposed to sub-minimal inhibitory concentration levels of biocides. Moreover, biofilm-forming microbes are often exposed to sublethal doses of antibiotics or disinfectants, since the biofilm structure generates a concentration gradient from the surface to their deeper parts [26].

The effects we observed were highly concentration dependent; at low concentration (0.1 mg·mL^−1^), the anti-adhesion activity of the fractions was much lower, or microbial adhesion even increased (especially with respect to nonpolar fractions). Fortunately, these unwanted adhesion-promoting effects were transient and did not decrease antibiofilm effectiveness after a longer co-incubation time of 24 h.

In general, antibiofilm activity of phenolic fractions obtained from all parts of the plant at 0.5× MIC (according to data given in Table 2) was stronger in relation to *C. albicans* than *S. aureus,* whereas the opposite tendency was found with the nonpolar preparations (Figure 1).

**Figure 2:**
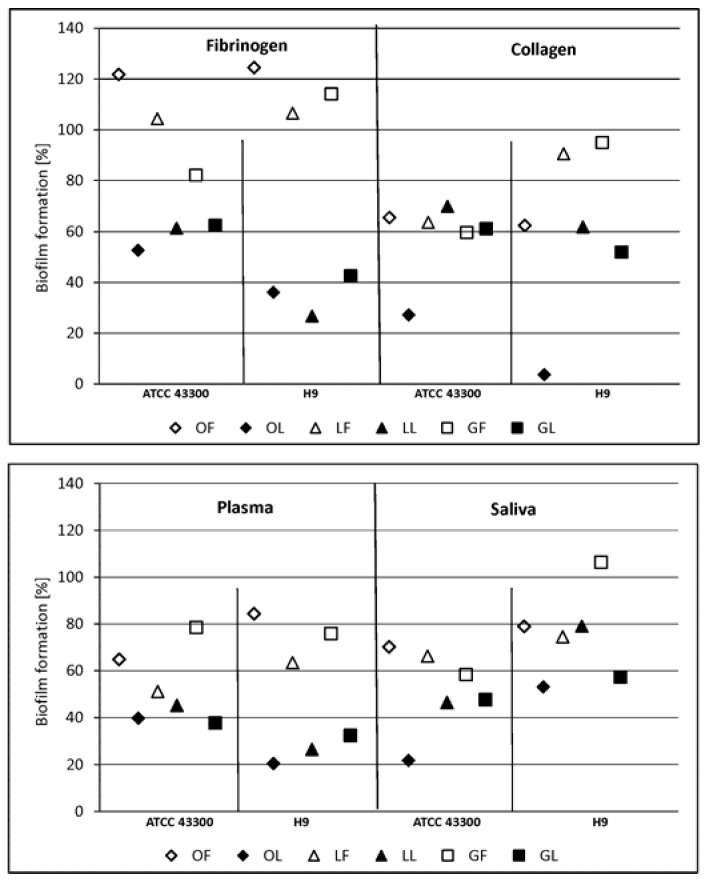
*Staphylococcus aureus* ATCC 43300 and clinical *S. aureus* H9 biofilm formation on the surface conditioned with host-derived proteins/body fluids (fibrinogen, collagen, plasma, saliva) in the presence of subMICs of fractionated sea buckthorn (SBT) extracts. LF, GF, OF denote phenolic fractions of leaf, twig, and fruit-derived extracts, respectively; LL, GL, OL denote nonpolar (lipid) fractions of leaf, twig, and fruit-derived extracts, respectively. Inhibitory effect was analyzed in terms of metabolic activity of biofilm mass by LIVE/DEAD BacLight Bacterial Viability kit (*S. aureus*). The percentage ± S.D. of biomass activity compared with the control (untreated), which was considered as 100%, is presented.

The higher efficiency of the nonpolar fraction at 0.1 mg·mL^−1^ against *S. aureus* was also seen when we examined the adhesion and biofilm formation on the surfaces conditioned with ECM proteins/glycoproteins. In the case of phenolic fractions, the weakest effect under these experimental conditions was noted with the surface coated with fibrinogen (Figure 2), which is unsurprising considering many more than one fibrinogen receptor is present on *S. aureus* cells as surface-anchored or secreted receptors.

Staphylococci express a broad range of surface proteins involved in their adhesion to ECM, plasma proteins, or directly to host cells. This binding capacity is closely related to their pathogenicity, adherence being a crucial step in the formation of biofilm and tissue invasion. As targets for staphylococci, fibrinogen, fibronectin, and collagen have the greatest significance during the process of infection. Collagen adhesins (CNA) allow bacteria to adhere strongly enough to tissue structures containing the corresponding ligand to resist clearance by the host defense system. The fibrinogen adhesins (specifically fibronectin binding protein - FnBPA/FnBPB, clumping factor - ClfA/ClfB, and several others with a wider substrate range) play a role in staphylococcal aggregation or “microcolony” formation—a process slightly different from classic biofilm formation. Examples of infections that may involve staphylococcal aggregates or microcolonies rather than typical biofilms include chronic wound infections, osteomyelitis, soft tissue abscesses, and endocarditis. In these cases, interactions with host matrix molecules are particularly important in colonization of the site, eukaryotic cell invasion by endocytosis, and evasion of an immune response [21,27,28]. Thus, limiting these interactions has great therapeutic potential, and our results with SBT-derived products fulfill these expectations.

Until now, research on *C. albicans* adherence has mainly addressed the three gene families *ALS*, *HWP*, and *IFF/HYR* encoding at least 25 adhesins of *C. albicans* with different spectra of ligands. However, a recent bioinformatics approach identified a plethora of proteins not previously implicated in adhesion and needing experimental confirmation of their significance. Among known candida adhesins, there are numerous receptors for plasma ECM proteins and saliva, as well as those ligands found on the surface of host cells [22,23,29,30,31,32,33]. We found the effect of inhibiting the formation of *C. albicans* biofilm by the components of SBT lipid fractions at low concentration (0.1 mg·mL^−1^) was poor, especially with respect to surfaces coated with collagen or with plasma. However, co-incubation of the yeast with phenolic fractions used at the same low concentration much more strongly inhibited biofilm formation, mainly on surfaces coated with fibrinogen or collagen.

**Figure 3:**
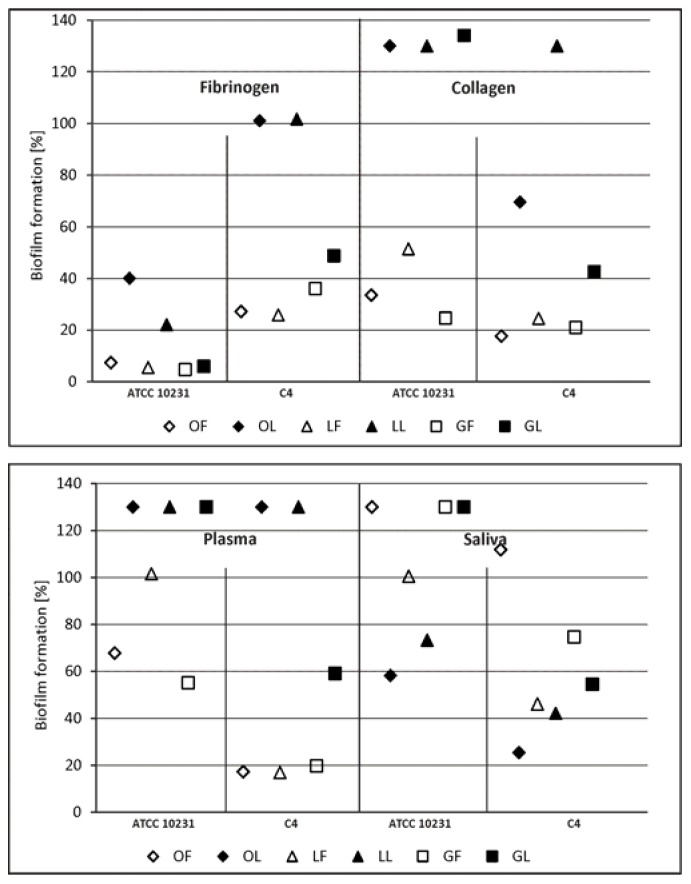
*Candida albicans* ATCC 10231 and clinical *C. albicans* C4 biofilm formation on surfaces conditioned with host-derived proteins/body fluids (fibrinogen, collagen, plasma, saliva) in the presence of subMICs of fractionated SBT extracts. LF, GF, OF denote phenolic fractions of leaf, twig, and fruit-derived extracts, respectively; LL, GL, OL denote nonpolar (lipid) fractions of leaf, twig, and fruit-derived extracts, respectively. Inhibitory effect was analyzed in terms of metabolic activity of biofilm mass by the “FDA reduction” method. The percentage ± S.D. of biomass activity compared with the control (untreated), which was considered as 100%, is presented.

For comparison, the same experiment was done with reference compounds present in the tested fractions. Individual compounds occurring in a leaf phenolic fraction (LF), such as ellagic acid and epicatechin present in the twig-derived phenolic fraction (GF), were less potent than the corresponding fraction type. For instance, in case of staphylococci on a collagen-conditioned surface, LF inhibitory activity reached 11.8% (Figure 2, no statistical significance), while there was no antiadhesive effect of ellagic acid. Adhesion of *C. albicans* to the collagen was inhibited by ellagic acid vs. LF up to 37.6% vs. 49.7% and 62.7% vs. 82.4% (Figure 3) for reference and clinical strain, respectively. According to phytochemical analysis of the SBT phenolic fractions, ellagic acid together with hydrolysable tannins and catechin together with proanthocyanidins make up to 31.3% and 47.5% of the composition of LF and GF, respectively (Table 1). Antimicrobial and antioxidant activity of tannins and ellagic acid, as well as an antiadhesive effect of proanthocyanidins were shown [1,3,7,14]. However, based on our results, the antiadhesive properties of LF and GF fractions seem to depend not only on the content of individual compounds, but the whole composition of such multi-ingredient preparations (possible synergistic activity of various compounds). Their antibiofilm activity varied, inhibiting biofilm formation in the range of 0–56.8%, which depended on both the type of microorganism and the type of proteins/glycoproteins deposited on the surface. A better result was obtained for staphylococci (up to 56.8% biofilm inhibition, *p* = 0.0004) than fungi (up to 27.3% inhibition, *p* = 0.24). Quercetin (a component of the phenolic fraction of the fruit extract) reduced both microbial adhesion and biofilm formation. *S. aureus* and *C. albicans* adhesion were inhibited, respectively, by 44.5–60.2% (*p* < 0.0009) and 0–86.4%. In the presence of quercetin, biofilm formation of *S. aureus* was restricted by 58.8–86.0% (*p* < 0.0014), and that of *C. albicans* by 47.7–90.7% (*p* < 0.03). It did not, however, have a strict impact on the activity of the OF fraction, which was practically ineffective against *S. aureus* adhesion and biofilm formation (Figure 2) and only partially capable of inhibiting *C. albicans* adherence and biofilm (Figure 3). In contrast, ursolic acid representing triterpenes, considered as a reference compound for all types of nonpolar fractions of SBT extracts, could have an influence on their activity. It was comparatively as effective as quercetin, reducing *S. aureus* biofilm by 44.3–94.6% (for most, *p* < 0.02) and that of *C. albicans* by 19.6–75.2% (for most, *p* < 0.03). Similar results were obtained for nonpolar fractions of SBT extracts, in particular, OL and GL, against staphylococcal biofilm (inhibitory effects up to 85.4% and 67.6%, respectively, Figure 2). It is worth emphasizing that triterpenes are the prevalent component of these factions (Table 1).

It should be emphasized that in experiments involving biofilm formation on surfaces conditioned with ECM proteins, SBT fractions were used at a low concentration of 0.1 mg·mL^−1^; and the antibiofilm effect was, in most cases, significant. It could be suggested that higher concentrations (0.25 or 0.5 mg·mL^−1^) of the phytopreparations might enhance such an effect. The results of the study on the effect of SBT extract used at 0.5 mg·mL^−1^ on microbial adhesion and biofilm formation on inert surfaces confirm such a possibility. However, such concentrations are rarely achieved in vivo. An excellent set of data on this topic can be found in the report by Manach et al. [34,35]. It should be noted, however, that the research on the metabolism of phytochemicals after oral intake and the concentrations achieved in the blood serum and tissues of internal organs concern supplementation mostly with products in their natural forms. Their chemical nature and routes of processing in the gastrointestinal tract determine the parameters of bioavailability and bioaccessibility, which commonly serve as references for predicting bioefficacy. Xiao et al. [36] have published a perspective paper in which edible nanoencapsulation vehicles (ENVs) for oral delivery of phytochemicals were discussed as bioefficacy enhancers. According to this literature review, ENVs influence the transportation of phytochemicals across the endothelial layer, enhancing paracellular transportation, opening tight junctions, strengthening mucosal adhesion, inhibiting efflux pumps, and inducing lymphatic absorption. Thus, ENVs can efficiently influence bioavailability and also exert an effect on phytochemical metabolism with the participation of the gut microbiota. Therefore, it can be assumed that the technological progress of ENV production will soon expand and improve the pharmacological use of phytochemicals.

#### 2.2.2. *C. albicans* Invasive Properties—Morphological Transformation

**Figure 4:**
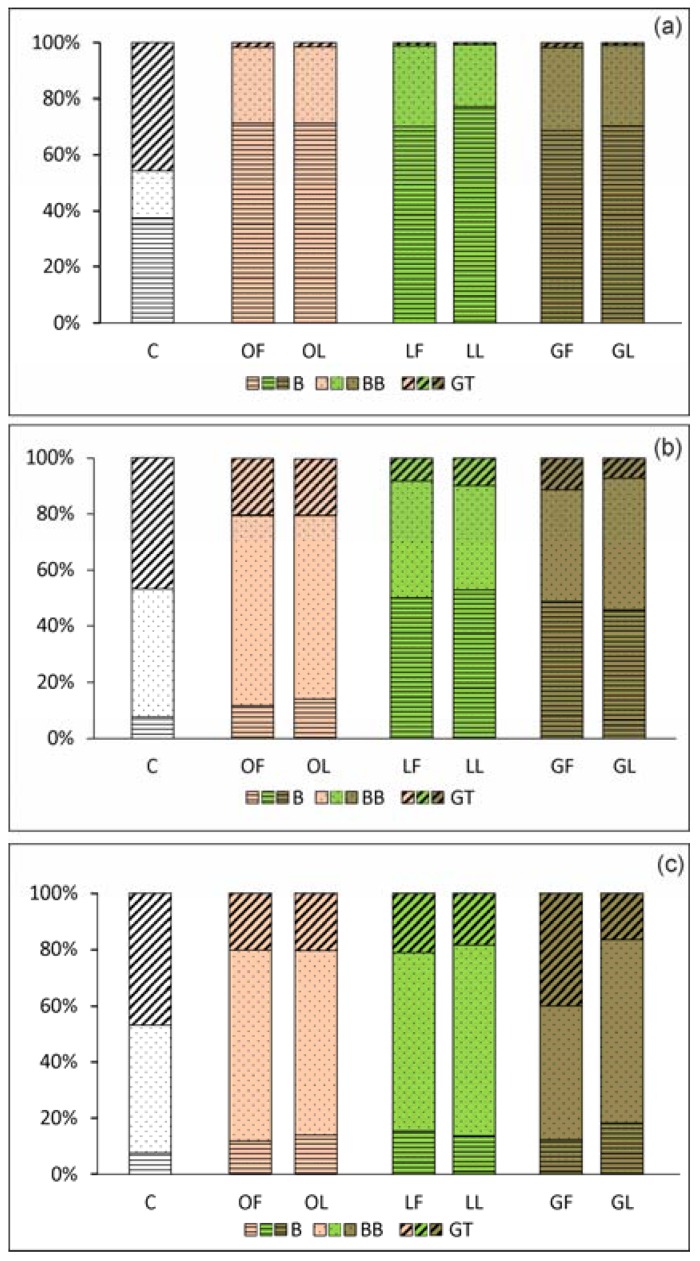
Percentage of *C. albicans* ATCC 10231 morphological forms (B, blastospores; BB, budding blastospores; GT, germ-tube-positive cells) after 1 (**a**), 2 (**b**), and 3 h (**c**) exposure to SBT fractions at 0.5× MIC. *C. albicans* cell morphology was examined by light microscopy (400× magnification) at these time points. The results are expressed as the proportion ± S.D. of each morphotype after SBT treatment, compared to control *C. albicans*, assessing 500 cells.

**Figure 5:**
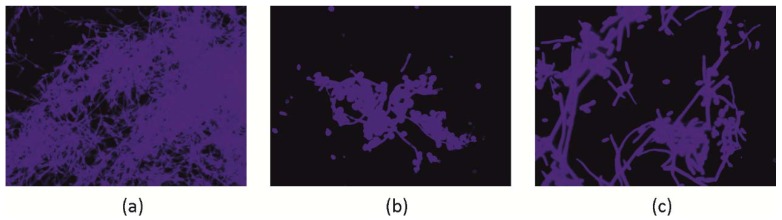
Mycelium of *C. albicans* ATCC10231 formation after 24 h exposure to SBT twig-derived fractions. (**a**) control; (**b**) GF, (**c**) GL at 0.5× MIC. *C. albicans* mycelium stained with Calcofluor White was checked microscopically (fluorescence microscope, Zeiss, AXIO Scope A1, magnification 400×); representative images are shown.

In the case of dimorphic fungi, interference in morphogenesis, i.e., transformation of blastospores through filaments (germ tubes, GT) up to real hyphae formation, is the most desirable property of a given natural product. Because both morphological forms play a role in *C. albicans* biofilm development, it means that these products can have therapeutic potential [22,24,29]. From experiments on the influence of SBT products on blastospore morphogenesis, a significant effect was achieved through the use of 0.5× MICs of the products. They reduce blastospore filamentation of *C. albicans* ATCC 10231, which progresses with the time of co-incubation. The formation of germ tubes after 1 h contact with SBT products was reduced by 50–65 times compared with control cells incubated in media containing only GT stimulating factors, i.e., serum (10%). This cell cycle “arrest” effect was maintained for the next hour, with about 5–8 times reduced morphogenesis occurring towards the formation of hyphae. High germ tube blocking activity of SBT was also maintained during the third hour of co-incubation. In the SBT-treated fungal cells, 16–20% of cells were GT-positive, whereas in the controls it was 46% (Figure 4). This conversion from yeast cells to hyphal growth seems to be one of the most prominent factors contributing to tissue invasion and resistance to phagocytosis. These forms also play a unique role in the process of *C. albicans* biofilm development by providing stability of the structure of the sessile population [22,23]. Interestingly, the reduction in the ability of *C. albicans* to form filaments was irreversible, as verified during prolonged co-culture for a total of 24 h when mycelium formation can be evaluated. The control culture in the optimal medium looked like densely entangled threads of hyphae, whereas in *C. albicans* cultures in the presence of phytocompounds, and regardless of their source (leaves, twigs, fruits), the fungi formed aggregates with few pseudohyphae and real hyphae. A representative microscopic image of such a culture is shown in Figure 5.

#### 2.2.3. *S. aureus* Invasive Properties—Adhesion to Monolayers of Eukaryotic Cells

Considering that staphylococci are etiologic agents of local and systemic infections, their interaction with fibroblasts and intestinal epithelial cells was examined in the presence of SBT-derived products, such as adhesion to a cell monolayer. This work was preceded, however, by an assessment of the biological safety of fractionated SBT extracts for the host cells (pro-proliferative activity/cytotoxicity). The results of an MTT test with HFF-1 fibroblasts showed that the fractionated SBT extracts at 0.007–1.0 mg·mL^−1^ did not reduce living cell numbers compared with control cells. IC_50_ values determined 24 h after exposure reached >1.0 mg·mL^−1^ for phenolic fractions of SBT fruit and twig extracts, and 0.865 mg·mL^−1^ for leaf extract. Nonpolar fractions yielded IC_50_ = 0.109, 1.394, and 0.236 mg·mL^−1^ for fruit, twig, and leaf extracts, respectively. This is encouraging for the future application of the preparations to eukaryotic tissues (e.g., as topically active ointments, lotions, or dressings).

Greater understanding is needed of the possibility of diminished bacterial adherence to and invasion into eukaryotic cells. Therefore, an anti-adhesion strategy can potentially be an alternative therapeutic means of overcoming the global threat of antibiotic resistance of *S. aureus*. These bacteria possess a number of adhesins allowing the above processes to occur; thus, the weakened adhesion to host cells achieved in our experiments is a real success, more so because this effect occurred at a relatively low concentration of the extracts (0.1 mg·mL^−1^), which can be achieved in vivo, e.g., by oral intake [34,35]. It is necessary, however, to explain that the anti-adhesive efficiency of all the tested fractions was not equal, but depended on the cell type (fibroblasts or intestinal epithelial cells) and the source of the extract. The phenolic fraction of the twig extract had the highest activity in this area as it decreased the adhesion of bacteria to a HFF-1 fibroblast monolayer by 7.3–9.8% and to a monolayer of Caco-2 intestinal epithelial cells by 19.7–32.4%. Nonetheless, we are convinced that the reduction of microbial adhesion by ~30% implies significance. The possibility is not excluded that the mechanism is through reducing the efficiency of sortases (SrtA, SrtB) responsible for the correct expression of surface adhesins. This is an important achievement because SrtA is now known to be a virulence factor of *S. aureus* that plays a major role in invasion and infection, whereas there are few reports concerning SrtB inhibitors [19,20,21,27,28].

### 2.3. Considerations on the Antimicrobial Activity of Fractionated SBT Extracts, in Relation to Their Composition

Analyzing the results of our research, we asked which fraction and/or its main component could be considered as the most promising product regarding therapeutic potential. Phenolic-rich fractions of fruit, leaf (supplementary Figure S1), and twig (supplementary Figure S2) extracts differed significantly—flavonoids, including quercetin, kaempferol, and a methylated metabolite of quercetin (isorhamnetin) have a quantitative advantage in fruit extracts, whereas hydrolysable tannins (ellagitannins) and triterpenoid saponins in the leaf extract and condensed tannins (proanthocyanidins, PACs) in twig extract dominated (Tables S1–S6). All of these chemical groups contain compounds that have been extensively studied in vitro regarding their antimicrobial activity. For example, Singh et al. [37] demonstrated that quercetin is a modulator of *C. albicans* quorum sensing (QS), which stimulates cell apoptosis and decreases fungal enzymatic activity, morphogenesis, and biofilm formation. Antibiofilm activity of quercetin and kaempferol against various bacterial species, including *S. aureus*, was also noticed [38,39]. Moreover, quercetin and isorhamnetin have been described as the compounds attenuating the virulence of *S. aureus*, causing down-regulation of the *agr* system, which consequently decreases synthesis of hemolysins [40].

Tannins are a heterogeneous group of polyphenolic compounds, naturally present in various plants, that exerts several pharmacological effects, including antimicrobial properties. Two different types can be distinguished: hydrolysable tannins (based on gallic acid and/or hexahydroxydiphenic acid, usually as multiple esters with d-glucose) present in the phenolic fraction of SBT leaf extract; and proathocyanidins, abundant in the fraction of twig extract. PACs are oligomeric or polymeric flavan-3-ols. They are divided into two classes—A-type and B-type—on the basis of the linkage among their monomeric units. Proanthocyanidins extracted from cranberry reduced biofilm formation by *S. mutans* in vitro and dental caries development in vivo due to the presence of specific bioactive A-type dimers and oligomers according to recent reports [41]. *C. albicans* adherence and biofilm formation were also inhibited by A-type PACs present in plant extracts [42,43,44]. Sea buckthorn seeds contain a substantial amount of proanthocyanidins, but little is known about their antimicrobial activity [45]. From our study it is now known that SBT twig extract is rich in PACs with B-type linkage, and that it influences bacterial and yeast behavior during multiplication, expression of cell-associated or secreted virulence factors, and all processes connected with biofilm formation.

Triterpenoid saponins present in phenolic and nonpolar fractions of SBT leaf extract are a diverse group of bioactive compounds possessing various activities, including antimicrobial, cell membrane perturbing, hemolytic, and cellular cytotoxicity [46]. However, our most interesting results concern the impact of the twig phenolic fraction components, such as the abovementioned PACs, and the components of the lipid fraction of the leaf extract containing mainly triterpenoids and acylated triterpenoids. In particular, pentacyclic triterpenoids, such as oleanolic and ursolic acid, are worthy of more attention as the constituents of numerous plants. Together they share many pharmacological properties, such as hepatoprotective effects, anti-inflammatory, antioxidant, or anticancer activities. Oleanolic acid, ursolic acid, α-amyrin, betulinic acid, and betulin aldehyde and other related triterpenoids are known to possess antimicrobial activity, which we also found in our explorations. It is important that some of these compounds, besides their direct antibacterial activity, have a synergistic effect in combination with antibiotics against multidrug-resistant pathogens and suppress bacterial virulence. The anti-staphylococcal properties of ursene and oleanene derivatives from *Castanea sativa* leaf extract reported by Cuave et al. [47] are of interest, showing an inhibitory effect against *S. aureus* and a panel of skin commensals. Ta et al. [5] published a review in which data on antibiofilm and anti-QS activity of plant secondary metabolites were collected.

## 3. Materials and Methods

### 3.1. Plant Material and Chemical Analysis of the Fractionated SBT-Derived Extracts

Sea buckthorn (*Elaeagnus rhamnoides* (L.) A. Nelson) branches were provided by a horticultural farm in Sokółka, Podlaskie Voivodeship, Poland. A voucher specimen (IUNG/HRH/2015/2) has been deposited at the Department of Biochemistry and Crop Quality, Institute of Soil Science and Plant Cultivation State Research Institute, Pulawy, Poland. The phenolic-rich and low-polarity fraction of the butanol extract from sea buckthorn (SBT) fruit was prepared and analyzed according to Olas et al. [11]. Preparation of butanol extracts from SBT leaves and twigs and their fractionation are described in details in the Supplementary Materials. Briefly, ground leaves and twigs of sea buckthorn were extracted with 80% methanol. The extracts were subjected to liquid–liquid extraction with *n*-butanol. The freeze-dried butanol extracts were suspended in 50% methanol, shaken, sonicated, and centrifuged. The supernatant containing mainly phenolic compounds was dried in a rotary evaporator, dissolved in 20% *tert*-butanol, and lyophilized. The pellet consisting mainly of less polar compounds was dissolved in methanol, rotary evaporated, dissolved in a mixture of *tert*-butanol and water, and lyophilized. Samples were analyzed using a Thermo Ultimate 3000 RS chromatographic system (Thermo Fischer Scientific, Waltham, MS, USA), equipped with a charged aerosol detector (CAD), a diode array detector (DAD), and coupled with a Bruker Impact II (Bruker Daltonics GmbH, Bremen, Germany) quadrupole-time of flight (Q-TOF) mass spectrometer. Chromatographic separations were performed on an ACQUITY BEH C18 column (2.1 × 150 mm, 1.7 µm; Waters, Milford, MA, USA), at 60 °C. UHPLC-ESI-MS analyses were carried out in negative and positive ion modes. Components of the analyzed fractions were identified on the basis of their HRMS and UV spectra, aided by data available in the literature.

### 3.2. Preparation of Solutions of Fractionated SBT-Derived Extracts and Reference Compounds

Stock solutions of fractionated SBT-derived butanol extracts were prepared in 100% methanol (phenolic fractions) or 100% DMSO (lipid fractions). Further dilutions of each stock were prepared in a medium adapted to the requirements of the test. Reference compounds, such as epicatechin, ellagic acid (both from Roth, Karlsruhe, Germany), quercetin, and ursolic acid (both from Sigma, Steinheim Germany) were dissolved in 100% DMSO and diluted in the appropriate medium. In some parts of the manuscript and Supplementary Materials (Tables and Figures), fractions were designated as LF, leaf/phenolic; LL, leaf/lipid; GF, twig/phenolic; GL, twig/lipid; OF, fruit/phenolic; OL, fruit/lipid.

### 3.3. Cytotoxic Activity of Fractionated SBT-Derived Extracts

Human fibroblasts (HFF-1, ATCC-SCRC-1041, LGC Standards, Poland) were grown in DMEM (Biowest, Riverside, MO, USA) containing high glucose, supplemented with 15% (*v*/*v*) heat-inactivated FBS (Biological Industries, Bet ha-Emek, Israel) and 1% (*v*/*v*) penicillin/streptomycin mixture (Biowest, USA), at 37 °C in a humidified atmosphere of air with 5% CO_2_ for 3 days. A confluent monolayer of the cells was detached with trypsin (Biowest, USA) and cell suspensions at 1 × 10^6^ cells·mL^−1^ were seeded into 96-well tissue culture plates (Nunc, Roskilde, Denmark) at 100 µL/well for 48 h incubation at 37 °C as above. The culture medium was replaced with 100 µL medium containing the fractionated SBT extracts over a concentration range of 0.007–1.0 mg·mL^−1^ for 24 h incubation, with appropriate positive and negative controls being set up at the same time. The pro-proliferative/cytotoxic activity of the fractions was measured by MTT [3-(4,5-dimethylthiazole-2-yl)-2,5-diphenyltetrazolium bromide] reduction assay [48]. Final absorbance of the samples was read at 550 nm with a Victor^2^ microplate reader (Wallac, Turku, Finland). The results for the test samples and the controls were used to calculate % cell viability and the IC_50_ (concentration giving 50% loss of viability).

### 3.4. Microorganisms and Culture Conditions

The reference/clinical 17 species/strains of bacteria and fungi belonging to the 9 following genera were used to screen the antimicrobial activity of fractionated SBT extracts: Gram-positive bacteria—*Staphylococcus*, *Streptococcus*, *Bacillus*, and *Lactobacillus*; Gram-negative bacteria*—Helicobacter*, *Pseudomonas*, *Escherichia*, and *Proteus*; and fungi—*Candida* (listed in Table 2). Microorganisms were grown for 24 or 48 h at 35–37 °C in media selected in accordance with their individual nutritional requirements; these were mainly TSB/TSA (tryptic soy broth/agar), SDA (Sabouraud’s dextrose agar), and RPMI-1640 medium. Suspensions of cells were prepared in a proper medium at a density required in each type of experiment to be carried out.

### 3.5. Minimal Inhibitory/Bactericidal/Fungicidal Concentration (MIC/MBC/MFC)

The MICs of phenolic and nonpolar fractions of SBT-derived extracts (from leaves, twigs, fruits), tested at a final concentration range of 0.0078–1.0 mg·mL^−1^, were determined by a broth microdilution method according to the EUCAST guidelines [49]. Briefly, the MIC was defined as the lowest concentration of the fraction inhibiting bacterial/fungal growth after 24–48 h of co-incubation at 35–37 °C compared with the appropriate positive controls. Solvents of fractionated SBT extracts—methanol and DMSO used at the highest final concentration in the medium (1.25% *v*/*v*)—served as controls, which did not disturb microorganism cell growth. The MBC/MFC of the preparations tested refers to the lowest concentration that prevented growth of the bacteria or yeast after subculturing 10 µL from the wells marked as MIC, 2× MIC, and 4× MIC in TSA/SDA medium, and after further incubation for 24 h at 35–37 °C. MICs of reference compounds, such as quercetin, epicatechin, ellagic acid, and ursolic acid (over a final concentration range of 0.0156–2.0 mg·mL^−1^), were determined by the same protocol. Experiments were carried out in duplicate in each of 2 separate sets of experiments.

### 3.6. S. aureus and C. albicans Adhesion and Biofilm Formation on the Abiotic (Polystyrene) Surface

The suspensions of *S. aureus* ATCC 43300 (reference, MRSA) and *S. aureus* H9 (clinical, MRSA) at a density of OD_535_ = 0.9 (~5 × 10^7^ cells·mL^−1^) in TSB/0.25% glucose; and *C. albicans* ATCC 10231 (reference, fluconazole (FLU) sensitive) and *C. albicans* C4 (clinical stool isolate, FLU sensitive) at 1 × 10^6^ cells·mL^−1^ in RPMI-1640/0.25% glucose were seeded (100 µL) into the wells of 96-well polystyrene culture microtiter plates (Nunc, Denmark). The fractionated SBT extracts at final concentrations of 0.125, 0.25, or 0.5 mg·mL^−1^ (corresponding to an earlier established value equal to 0.5× MIC with respect to a given strain) were added (100 µL). An additional concentration tested was 0.1 mg·mL^−1^ (the reason being explained in the Results section). To measure staphylococcus or yeast adhesion, samples were incubated at 37 °C in static conditions for 1 or 2 h, respectively; to measure biofilm formation, the incubation time was prolonged to 24 h. Microbial suspensions in medium (100 µL:100 µL) and medium alone (200 µL) served as the positive and negative controls, respectively. After incubation, at the indicated time point, the nonadherent cells were removed by washing the wells with 200 μL PBS with Ca^2+^ and Mg^2+^ (Biowest, USA) and the viability or metabolic activity of the sessile population was measured. In the case of *S. aureus*, a LIVE/DEAD BacLight Bacterial Viability kit (Molecular Probes, Eugene, OR, USA) was used. Finally, the fluorescence in the wells was measured at 485_ex_/535_em_ nm for green Syto9 and at 485_ex_/620_em_ nm for red PI, using SpectraMax i3 Molecular Devices. The results are given as a percentage of adherent cells or biofilm biomass calculated from the mean fluorescence values ± S.D. of the control wells containing bacteria in medium without SBT (taken as 100%) and of the test wells. For *C. albicans*, a self-modified “FDA reduction” method was used as previously described [6]. Briefly, 100 µL FDA (fluorescein diacetate, Sigma, St. Louis, MO, USA) solution (0.2 mg·mL^−1^ in phosphate buffer, pH 6.8) was added to the wells for 1 h incubation at 37 °C in the dark and the fluorescence emitted was read at 485_ex_/520_em_ nm using SpectraMax i3 Molecular Devices. The results are given as the percentage of adherent cells or total biomass metabolic activity, calculated from the RFU (relative fluorescence units) values ± S.D. in the test wells compared with the controls (taken as 100%). The experiments were carried out twice in quadruplicate.

### 3.7. S. aureus and C. albicans Adhesion and Biofilm Formation on the Surface Conditioned with Host-Derived Proteins/Body Fluids

Surfaces conditioned with proteins (fibrinogen, collagen) or body fluids (plasma and saliva) involved 96-well microplates as follows: (i) microplate wells coated on their own with fibrinogen (Sigma, Germany) 20 µg·mL^−1^ in carbonate buffer, pH 9.6 (Na_2_CO_3_, 1.59 g·L^−1^; NaHCO_3_, 2.93 g·L^−1^), 100 µL/well; (ii) commercial Cell Culture Microplate, with Cellcoat Collagen type I (Greiner, Frickenhausen, Germany); (iii) microplate wells coated on their own with 100% human plasma (Biowest, USA), 100 µL/well; (iv) microplate wells coated on their own with artificial saliva with the composition mucin (Roth, Germany), 3.5 g; K_2_HPO_4_, 57 mg; CaCl_2_, 11 mg; MgCl_2_ × 6H_2_O, 17 mg; KCl, 75 mg; K_2_CO_3_, 53 mg; NaCl, 33 mg (all from POCH, Gliwice, Poland); H_2_O, deionized, 100 mL (Sigma, Germany), pH = 6.8, 100 µL/well. Steps and conditions of coating: 18 h at 4 °C; removal of proteins/body fluids; blocking of tested and control (uncoated) wells with 250 µL/well 2% bovine serum albumin (BSA, Sigma, USA) in PBS for 18 h at 4 °C; washing once with PBS. The next stages of the experiment regarding the application of microorganisms, tested SBT fractions at low concentration (0.1 mg·mL^−1^), co-incubation conditions, and the method of evaluating and interpreting the results were the same as those described in the previous section. The wells containing only bacterial or fungal suspensions in the culture medium (without SBT) were taken as the positive control (100%). The experiments were repeated twice with 6 replications of each.

### 3.8. C. albicans Invasive Properties—Evaluation of Morphogenesis Potential

To determine the serum-induced filamentation of fungi, a previously described microscopic method was used. Briefly, *C. albicans* ATCC 10231 and *C. albicans* A4 suspensions (8 × 10^6^ blastospores·mL^−1^) in RPMI-1640 without phenol red and supplemented with 10% (*v*/*v*) FBS (Biological Industries, Israel) were incubated without (control) or with the addition (1:1) of fractionated SBT-derived extracts (final concentrations 0.5, 1.0 mg·mL^−1^) for 1, 2, or 3 h at 37 °C. At indicated time points, the number of germ tube forms, budding cells, and intact blastospores was estimated by light microscopy (Nikon Eclipse E200, at magnifications of 400× or 200×, depending on morphogenesis progress). The results have been expressed as a percentage of each cell morphotype in samples treated with SBT products and control (calculated from total 500 cells counted ± S.D). The criteria adopted for the morphogenesis were as described elsewhere [6]. For comparison, reference compounds were used under the same experimental protocol. After prolonged (an additional 24 h) incubation time, hyphal morphology was assessed microscopically by staining the developed mycelium with ready-to-use Calcofluor White solution (Sigma, USA) as described [6]. Results were assessed microscopically (fluorescence microscope, Zeiss, AXIO Scope A1, magnification 400×) and representative images were taken for analysis.

### 3.9. S. aureus Invasive Properties—Evaluation of Adhesion to Eukaryotic Cell Monolayers

Bacteria from fresh culture at 1 × 10^8^ cells·mL^−1^ were labeled by incubation at room temperature for 20 min with 1 mg·mL^−1^ of fluorescein isothiocyanate (FITC, isomer I, Sigma, USA) in PBS, as per Sadowska et al. [50]. Labeled bacteria were finally suspended in cell culture medium without antibiotics and used in the adherence assay. HFF-1 (ATCC-SCRC-1041) and Caco-2 (ATCC-HTB-37) cell lines were grown to reach semiconfluent monolayers. For Caco-2 cells, DMEM medium without sodium pyruvate (Biowest, USA) was used containing high glucose, HEPES, 10% FBS (not inactivated; Biological Industries, Israel), and 1% penicillin/streptomycin (Biowest, USA). The wells were blocked by 2% BSA in PBS (20 min, 37 °C, air plus 5% CO_2_). Samples of FITC-labeled bacteria (100 µL) and 100 µL SBT fractions at a final concentration of 0.1 mg·mL^−1^ were added (in 6 repetitions) and incubated for 1 h at 37 °C in 5% CO_2_ in air. Appropriate controls (cells in medium, bacteria alone over a range of dilutions, to obtain the standard curve) were prepared each time. Nonadherent bacteria were removed by aspiration, and PBS was added (200 µL/well). The fluorescence of the monolayer–bacterium complex was measured on a Victor^2^ multifunctional counter (Wallac, Turku, Finland). The percentage of fluorescence reflecting the degree of bacterial adherence was calculated, taking into account the appropriate (positive and negative) control measurements.

### 3.10. Statistical Analysis

Statistical differences were evaluated using the Mann–Whitney *U* test or Kruskal–Wallis one-way ANOVA. STATISTICA 12.0 (Stat Soft Inc., Palo Alto, CA, USA) software was used for the calculations. A value *p* ≤ 0.05 was considered statistically significant. Some results are provided as mean values with the corresponding standard deviations (S.D.).

## 4. Conclusions

Fractionated sea-buckthorn-derived extracts have relatively weak biostatic/biocidal activity against bacteria and fungi, lower than their starting products (butanol extracts of leaves, twigs, and fruits). However, they attenuate essential pathogenic properties of *S. aureus* and *C. albicans* when used at subMIC. They decreased adhesion and biofilm formation on inert surfaces and also on surfaces conditioned with fibrinogen, collagen, plasma, or saliva. They negatively influenced the invasive properties of *S. aureus* (adhesion to a monolayer of fibroblasts and intestine epithelium cells), as well as invasion-associated morphogenesis progress of *C. albicans* (germ tubes and mycelium formation). These effects were dependent on both the type of fraction (phenolic or nonpolar) and the origin of their extracts (leaves, twigs, and fruits). Knowing the phytochemical composition of the extracts it can be speculated which component may be responsible for the observed effect. However, in the case of phenolic fractions of SBT extracts with a more diverse composition, there was no such direct relation compared to the activity of the reference compounds. We can rather expect either synergistic or antagonistic effect between various components of such multi-ingredient preparations. The new information afforded by this study, additional to that already known, is of potential practical value in the application of SBT-derived preparations as antivirulence agents. More detailed studies are needed to gain a better understanding of the mechanisms of action and safety–composition relationships of complex products of this nature.

## Figures and Tables

**Table 1:** The relative content of selected individual groups of compounds in the phenolic and non-polar fractions of leaf, twig and fruits of sea buckthorn extracts, expressed as a percentage of total peak area (Corona charged Aerosol Detector). Values above 5% are presented.

Fraction	Compound Type	Relative Peak Area (%)		
		Leaf	Twig	Fruit *
Phenolic	Unidentified polar compounds	15.8	35.7	20.9
	Hydrolysable tanins and ellagic acid	31.3	-	-
	Proanthocyanidins and catechin	-	47.5	-
	Isorhamnetin glycosides	7.0	-	29.5
	Triterpenoid saponins	15.0	-	-
	Triterpenoids	5.8	5.4	8.0
Non-polar	Unidentified non-polar compound	18.9	36.5	29.7
	Triterpenoid saponins	30.5	-	-
	Triterpenoids	38.5	33.9	44.8
	Acylated triterpenoids **	5.6	24.4	24.5

* data published in Food Chem., 2018; ** acylated with phenolic acids.

**Table 2:** The antimicrobial activity of fractionated Sea buckthorn extracts. Minimal inhibition concentration (MIC) measured by broth microdilution assay.

Microorganism	Phenolic Fraction (MIC) [mg∙mL^−1^]	
	LF (Leaf Extract)	GF (Twig Extract)
**Gram-positive**		
*Staphylococcus aureus* ATCC 29213	0.5	1.0
*Staphylococcus aureus* ATCC BAA-1708	0.5	0.5
*Staphylococcus aureus* ATCC 43300	0.5	0.25
*Staphylococcus aureus* H9 *	0.5	0.5
*Streptococcus mutans* ATCC 25175	>1.0	>1.0
*Bacillus cereus* ATCC	0.5	0.5
*Lactobacillus acidophilus* ATCC 4356	>1.0	>1.0
**Gram-negative**		
*Helicobacter pylori* ATCC 700392	1.0	1.0
*Escherichia coli* ATCC 25922	>1.0	>1.0
*Proteus vulgaris* ATCC 8427	1.0	1.0
*Pseudomonas aeruginosa* ATCC 25619	>1.0	>1.0
**Fungi**		
*Candida albicans* ATCC 10231	1.0	1.0
*Candida albicans* ATCC 90028	>1.0	>1.0
*Candida albicans* A4 *	>2.0	>1.0
*Candida parapsilosis* ATCC 22019	1.0	1.0
*Candida krusei* ATCC 14243	>1.0	>1.0
*Candida glabrata* G1 *	>1.0	>1.0

* clinical strain.

**Table 3:** The antimicrobial activity of reference phytochemicals. Minimal inhibition concentration (MIC) measured by broth microdilution assay.

Microorganism	Compound (MIC) [mg∙mL^−1^]			
	Ellagic Acid	Epicatechin	Quercetin	Ursolic Acid
*Staphylococcus aureus* ATCC 43300	>1.0	>1.0	>1.0	0.25
*Staphylococcus aureus* H9 *	>1.0	1.0	>1.0	0.5
*Candida albicans* ATCC 10231	2.0	>2.0	>2.0	0.031
*Candida albicans* A4 *	>2.0	>2.0	>2.0	0.031

* clinical strain.

## Supplementary Materials

The following are available online. Details on plant material, preparation, analysis, and chemical characterization of fractions, discussion, literature. Tables S1–S6; Figures S1 and S2. Table S1. The relative content of individual groups of compounds in the phenolic fraction (LF) and in the nonpolar fraction (LL) of sea buckthorn leaf extract, expressed as a percentage of the total peak area (Corona Charged Aerosol Detector). Table S2. The relative content of individual groups of compounds in the phenolic fraction (GF) and in the nonpolar fraction (GL) of sea buckthorn twig extract, expressed as a percentage of the total peak area (Corona Charged Aerosol Detector). Table S3. Secondary metabolites in the phenolic-rich fraction of sea buckthorn leaf extract (LF); the listed compounds correspond to UHPLC-CAD peaks with area ≥1% of the total peak area. Table S4. Secondary metabolites in the nonpolar fraction of sea buckthorn leaf extract (FL); the listed compounds correspond to UHPLC-CAD peaks with area ≥1% of the total peak area. Table S5. Secondary metabolites in the phenolic-rich fraction of sea buckthorn twig extract (GF); the listed compounds correspond to UHPLC-CAD peaks with area ≥1% of the total peak area. Table S6. Secondary metabolites in the nonpolar fraction of sea buckthorn twig extract (GL); the listed compounds correspond to UHPLC-CAD peaks with area ≥1% of the total peak area. Figure S1. UHPLC-CAD chromatograms of the phenolic-rich fraction LF (A) and the nonpolar fraction LL (B) from *E. rhamnoides* (L.) A. Nelson leaves. Figure S2. UHPLC-CAD chromatograms of the phenolic-rich fraction GF (A) and the nonpolar fraction GL (B) from *E. rhamnoides* (L.) A. Nelson twigs.
