# Supplementary material for: Phenolic and Nonpolar Fractions of Elaeagnus rhamnoides (L.) A. Nelson Extracts as Virulence Modulators—In Vitro Study on Bacteria, Fungi, and Epithelial Cells
Source: Molecules. 2018 Jun 21;23(7):1498. doi: 10.3390/molecules23071498 (PMC6099406; doi:10.3390/molecules23071498)
Supplement: Supplementary file 1 [file molecules-23-01498-s001.pdf]

SUPPLEMENTARY MATERIALS

Phenolic and Nonpolar Fractions of *Elaeagnus rhamnoides* (L.) A. Nelson Extracts as Virulence Modulators—In Vitro Study on Bacteria, Fungi, and Epithelial Cells

Barbara Różalska[1*], Beata Sadowska[1], Jerzy Żuchowski[2], Marzena Więckowska-Szakiel[1], Aleksandra Budzyńska[3], Urszula Wójcik[1], Anna Stochmal[2]

[1]Department of Immunology and Infectious Biology, Institute of Microbiology, Biotechnology and Immunology, Faculty of Biology and Environmental Protection, University of Lodz, Banacha 12/16, 91-237 Lodz, Poland,

[2]Department of Biochemistry, Institute of Soil Science and Plant Cultivation, State Research Institute, Czartoryskich 8, 24-100 Pulawy, Poland,

[3]Laboratory of Microbiological and Technical Services; Institute of Microbiology, Biotechnology and Immunology, Faculty of Biology and Environmental Protection, University of Lodz, Banacha 12/16, 91-237 Lodz, Poland

[*] Correspondence: e-mail: barbara.rozalska@biol.uni.lodz.pl Phone:+48 42 635 41 85

1. Plant Material

Sea buckthorn—*Elaeagnus rhamnoides* (L.) A. Nelson (*Hippophae rhamnoides* L.) branches were provided by a horticultural farm in Sokółka, Podlaskie Voivodeship, Poland. A voucher specimen (IUNG/HRH/2015/2) has been deposited at the Department of Biochemistry and Crop Quality, Institute of Soil Science and Plant Cultivation State Research Institute, Puławy, Poland.

1.1. Preparation of Extracts from Sea Buckthorn Leaves and Twigs

Freeze-dried sea buckthorn (SBT) leaves and twigs (twigs air-dried at 40 ˚C) were milled in a laboratory mill (Retsch ZM200, Germany) and were stored in a freezer. The powdered leaves (284 g) were extracted with 5 L (3 portions) of 80% methanol (v/v), for 48 h, at room temperature; the extraction was assisted by ultrasonication (3 × 10 min). The milled twigs (680 g) were extracted with 14 L of 80% methanol (3 portions). After filtration, the extracts were concentrated by rotary evaporation (40 ˚C), and extracted with *n*-hexane. Organic solvents were removed in a rotary evaporator, the residue was subsequently resuspended in Milli-Q water (final volume ~1200 mL) and subjected to *n*-butanol extraction (200 mL portions). The obtained butanol extracts were rotary evaporated to remove the solvent; the residue was suspended in Milli-Q water (small portions of 20% *tert*-butanol solution were additionally used to dissolve the sediment from the evaporation flask) and freeze-dried. The used procedure yielded 12.42 g of the dry leaf extract and 24.64 g of the twig extract. A 12 g portion of the butanol extract of SBT leaves was suspended in 600 mL of 50% methanol, shaken, sonicated for 2 min, and centrifuged. The supernatant, containing mainly phenolic compounds, was dried in a rotary evaporator, dissolved in 20% *tert*-butanol, and freeze-dried, to yield 11.37 g of the phenolic-rich fraction. The pellet, which consisted mainly of less polar compounds, was dissolved in methanol and rotary evaporated. The sediment was dissolved in a mixture of *tert*-butanol and water, and freeze-dried (0.63 g). The same method of fractionation was applied for the twig extract (14 g was mixed with 700 mL of 50% methanol), yielding finally 13.07 g of the phenolic-rich fraction, and 0.83 g of the low-polarity fraction of the twig butanol extract.

LC-MS

Samples were analyzed using a Thermo Ultimate 3000RS (Thermo Fischer Scientific, Waltham, MS, USA) chromatographic system, equipped with a charged aerosol detector (CAD), a diode array detector (DAD), and coupled with a Bruker Impact II (Bruker Daltonics GmbH, Germany) quadrupole-time of flight (Q-TOF) mass spectrometer. Separations of samples were performed on a Waters BEH C18 column (2.1 × 150 mm, 1.7 µm; Milford, MA, USA) at 60 °C. The injection volume was 2.5 µL. The mobile phase A was 0.1% (v/v) formic acid in MilliQ water, the mobile phase B was acetonitrile containing 0.1% (v/v) of formic acid. Chromatographic separations (500 $\mu L \cdot min^{-1}$, 30 min) were carried out using a linear gradient from 7 to 90% of solvent B in solvent A. UHPLC-ESI-MS analyzes were performed in negative and positive ion mode. The scanning was set from *m*/*z* 50 to *m*/*z* 2000. The following MS settings were applied for negative ion mode: capillary voltage 3 kV; dry gas flow 6 $L \cdot min^{-1}$; dry gas temperature 200 °C; nebulizer pressure 0.7 bar; collision RF 700 Vpp; transfer time 80 µs; prepulse storage time 10 µs. Collision energy was set automatically in the range from 15 to 140 eV, depending on the *m*/*z* of a fragmented ion. MS settings for positive mode: capillary voltage 4.5 kV; dry gas flow 6 $L \cdot min^{-1}$; dry gas temperature 200 °C; nebulizer pressure 0.7 bar; collision RF 700 Vpp; transfer time 70 µs; prepulse storage time 7 µs. Collision energy was set automatically in the range from 9 to 85 eV, depending on the *m*/*z* value of a fragmented ion.

Components of the analyzed fractions were tentatively identified on the basis of their HRMS and UV spectra, with a help of available literature data. Four flavonoids were more precisely identified by comparison with retention times of standards. The relative content of individual groups of compounds was evaluated on the basis of CAD chromatograms and expressed as a percentage of the total peak area.

1.2. Preparation and Analysis of Fractions from Sea Buckthorn Fruits

The phenolic-rich (OF) and low-polarity (OL) fractions of the butanol extract from sea buckthorn fruits were prepared and analyzed as described by Olas et al. [1]. UHPLC-MS analyses of the preparations demonstrated that flavonol glycosides and acylated flavonol glycosides were dominant compounds of OF: their relative content was 39.5% and 27.6% of the total peak area for simple and acylated flavonoids, respectively. It contained also some unidentified polar (20.9%) and nonpolar compounds (2.4%), as well as triterpenoids (8%) and acylated triterpenoids (1.1%). The fraction OL contained mainly triterpenoids (44.8% of the total peak area), acylated triterpenoids (24.5%), and unidentified nonpolar compounds (29.7%), with a small addition of flavonoids and unidentified polar compounds (1% in total).

2. Results

2.1. Chemical Characterization of the Sea Buckthorn Fractions

Hydrolysable tannins, represented mainly by different types of ellagitannins, were dominant constituents of the phenolic-rich fraction of sea buckthorn leaves (LF). Hydrolysable tannins, together with small amounts of ellagic acid, constituted 31.3 % of the total peak area (**Table S1**, **Figure S1**). Flavonoids were the second most abundant group of phenolic compounds, constituting 24.5 % of the total peak area. They were glycosides of isorhamnetin, quercetin, and kaempferol, both simple and acylated. Kaempferol hexosides acylated with *p*-coumaric acid and isorhamnetin diglycosides acylated with rarely occurring (putative) linalool-1-oic acid were dominant acylated flavonoids. The preparation contained also significant amounts of unidentified polar compounds and triterpenoid saponins (with aglycones having formulas $C_{30}H_{48}O_4$, $C_{30}H_{46}O_4$, as well as $C_{30}H_{48}O_3$). The fraction contained also some triterpenoids and acylated triterpenoids, as well as unidentified nonpolar compounds **(Table S3**). Unsurprisingly, the low-polarity fraction (LL) was composed mostly of hydrophobic compounds (**Table S1**, **Table S4, Figure S1**), mainly triterpenoids and triterpenoid saponins, with smaller amounts of unidentified hydrophobic compounds and acylated triterpenoids. The preparation contained also small portions of ellagitannins, flavonoids, and unidentified polar compounds.

B-type proanthocyanidins (37.5 % of the total peak area) and catechin (10.0 %) were major constituents of the phenolic-rich fraction of sea buckthorn twigs (GF). The fraction had a high content of unidentified polar substances; it contained also small amounts of flavonoids, ellagitannins, and ellagic acid, as well as triterpenoids, acylated triterpenoids, and unidentified nonpolar compounds (**Table S2**, **Table S5**, **Figure S2**). In contrast, the low-polarity fraction of the twig extract (GL) consisted mainly of triterpenoids and acylated triterpenoids; it also had a significant share of unidentified nonpolar compounds (**Table S2**, **Table S6, Figure S2**). The fraction contained also small amounts of proanthocyanidins, catechin, and unidentified polar compounds .

Table S1. The relative content of individual groups of compounds in the phenolic fraction (LF) and in the nonpolar fraction (LL) of sea buckthorn **leaf** extract, expressed as a percentage of the total peak area (Corona Charged Aerosol Detector).

	Relative peak area (%)	Dominant compounds
	LF	
Unidentified polar compounds	15.8	
Gallocatechin	1.6	
Hydrolysable tannins and ellagic acid	31.3	stachyurin, casuarinin hippophaenin B or isomers
Kaempferol glycosides	0.7	K-dHex-Hex, K-Hex
Acylated kaempferol glycosides	4.1	K-Hex-pCou
Quercetin glycosides	4.0	Q-3-*O*-Glc, rutin
Acylated quercetin glycosides	1.8	Q-Hex-dHex-166
Isorhamnetin glycosides	7.0	I-3-*O*-Glc-7-*O*-Rha, I-rutinoside
Acylated isorhamnetin glycosides	6.9	I-dHex-Hex-166
Unidentified nonpolar compounds	4.2	
Triterpenoid saponins	15.0	$C_{71}H_{112}O_{31}$, $C_{69}H_{110}O_{29}$
Triterpenoids	5.8	$C_{30}H_{48}O_5$, $C_{30}H_{48}O_4$
Acylated triterpenoids*	1.8	$C_{39}H_{54}O_7$
	LL	
Unidentified polar compounds	1.2	
Hydrolysable tannins	2.7	stachyurin, casuarinin hippophaenin B or isomers
Kaempferol glycosides	0.4	K-dHex-Hex
Acylated kaempferol glycosides	0.6	K-Hex-pCou
Quercetin glycosides	0.1	rutin
Acylated quercetin glycosides	0.3	Q-Hex-dHex-166
Isorhamnetin glycosides	0.6	I-3-*O*-Glc-7-*O*-Rha, I-rutinoside
Acylated isorhamnetin glycosides	0.6	I-dHex-Hex-166
Unidentified nonpolar compounds	18.9	
Triterpenoid saponins	30.5	$C_{69}H_{110}O_{29}$, $C_{63}H_{100}O_{25}$, $C_{57}H_{90}O_{20}$, $C_{63}H_{100}O_{24}$
Triterpenoids	38.5	$C_{30}H_{48}O_5$, $C_{30}H_{48}O_4$
Acylated triterpenoids*	5.6	$C_{39}H_{54}O_7$, $C_{39}H_{54}O_6$

I – isorhamnetin; K – kaempferol; Q – quercetin; dHex – deoxyhexose; Hex – hexose; Glc – glucose; Rha – rhamnose; pCouA – *p*-coumaric acid; FerA – ferulic acid; 166 – linalool-1-oic acid

*Acylated with phenolic acids

Table S2. The relative content of individual groups of compounds in the phenolic fraction (GF) and in the nonpolar fraction (GL) of sea buckthorn **twig** extract, expressed as a percentage of the total peak area (Corona Charged Aerosol Detector).

	Relative peak area (%)	Dominant compounds
	GF	
Unidentified polar compounds	35.7	
Gallocatechin	1.6	
Hydrolysable tannins and ellagic acid	1.9	ellagic acid, stachyurin & casuarinin or isomers
Proanthocyanidins and catechin	47.5	catechin, dimeric & trimeric proanthocyanidins
Acylated kaempferol glycosides	0.4	K-Hex-pCou
Isorhamnetin glycosides	1.3	I-rutinoside, I-dHex
Acylated isorhamnetin glycosides	0.6	I-dHex-Hex-Hex-FerA
Unidentified nonpolar compounds	4.3	
Triterpenoids	5.4	$C_{30}H_{48}O_5$, $C_{30}H_{48}O_4$
Acylated triterpenoids*	1.3	$C_{39}H_{54}O_7$, $C_{39}H_{54}O_6$
	GL	
Unidentified polar compounds	3.8	
Proanthocyanidins and catechin	1.3	catechin, dimeric & trimeric proanthocyanidins
Unidentified nonpolar compounds	36.5	
Triterpenoids	33.9	$C_{30}H_{48}O_5$, $C_{30}H_{48}O_4$
Acylated triterpenoids*	24.5	$C_{39}H_{54}O_7$, $C_{39}H_{54}O_6$

I – isorhamnetin; K – kaempferol; dHex – deoxyhexose; Hex – hexose; FerA – ferulic acid;
*Acylated with phenolic acids

Table S3. Secondary metabolites in the phenolic-rich fraction of sea buckthorn **leaf** extract (LF); the listed compounds correspond to UHPLC-CAD peaks with area ≥1% of the total peak area.

No.	t_R [min]	$[M-H]^-$ (*m*/*z*)	$[M-H]^-$ formula	Tentative identification	Relative peak area (%)	Ref.
1	1.0	331.0665	$C_{13}H_{15}O_{10}$	GalA-Hex	2.1	
2	1.3	305.0666	$C_{15}H_{13}O_7$	(epi)gallocatechin	1.6	
3	2.3	633.0734	$C_{27}H_{21}O_{18}$	strictinin or isomer	5.2	[2,3]
4	2.5	935.0785	$C_{41}H_{27}O_{26}$	stachyurin, casuarinin	10.3	[2,3]
		1103.0858	$C_{48}H_{31}O_{31}$	hippophaenin B or isomers		
5	3.2	785.0843	$C_{34}H_{25}O_{22}$	ellagitanin	1.5	[2,3]
6	3.8	935.0793	$C_{41}H_{27}O_{26}$	casuarictin or isomer	3.4	[2,3]
7	4.1	1117.0998	$C_{49}H_{35}O_{31}$	ellagitannin	1.6	
8	4.6	1085.0731	$C_{46}H_{29}O_{30}$	ellagitannin	1.4	
9	4.8	300.9982	$C_{14}H_5O_8$	ellagic acid	1.2	
10	5.4	609.1451	$C_{27}H_{29}O_{16}$	Q-3-*O*-rutinoside	1.3	[4]
11	5.5	463.0868	$C_{21}H_{19}O_{12}$	Q-3-*O*-Glc	1.3	[4]
12	5.7	623.1604	$C_{28}H_{31}O_{16}$	I-dHex-Hex	1.8	
13	5.9	623.1606	$C_{28}H_{31}O_{16}$	I-3-*O*-Glc-7-*O*-Rha	2.0	[4]
14	6.4	961.2606	$C_{44}H_{49}O_{24}$	I-dHex-Hex-Hex-FerA	1.1	
15	6.8	623.1607	$C_{28}H_{31}O_{16}$	I-3-*O*-rutinoside	1.1	[4]
16	9.8	593.1298	$C_{30}H_{25}O_{13}$	K-Hex-pCouA	2.3	[4]
17	10.3	593.1297	$C_{30}H_{25}O_{13}$	K-Hex-pCouA	1.2	[4]
18	12.6	789.2606	$C_{38}H_{45}O_{18}$	I-dHex-Hex-166	1.0	[4]
19	12.7	789.2602	$C_{38}H_{45}O_{18}$	I-dHex-Hex-166	1.9	[4]
20	13.3	1235.6061	$C_{59}H_{95}O_{27}$	triterpenoid saponin	1.2	
21	14.6	1381.6639	$C_{65}H_{105}O_{31}$	triterpenoid saponin	1.1	
22	16.2	1219.6106	$C_{59}H_{95}O_{26}$	triterpenoid saponin	1.2	
23	16.5	1459.7109	$C_{71}H_{111}O_{31}$	triterpenoid saponin	2.1	
24	16.9	1313.6519	$C_{65}H_{101}O_{27}$	triterpenoid saponin	1.6	
25	17.7	1401.7059	$C_{69}H_{109}O_{29}$	triterpenoid saponin	2.1	
26	17.8	1297.6559	$C_{65}H_{101}O_{26}$	triterpenoid saponin	1.6	
27	18.5	487.3418	$C_{30}H_{47}O_5$	triterpenoid	3.5	
28	19.5	1239.6523	$C_{63}H_{99}O_{24}$	triterpenoid saponin	1.0	
29	23.0	471.3474	$C_{30}H_{47}O_4$	triterpenoid	1.3	

I – isorhamnetin; K – kaempferol; Q – quercetin; dHex – deoxyhexose; Hex – hexose; Glc – glucose; Rha – rhamnose; GalA – gallic acid; pCouA – *p*-coumaric acid; FerA – ferulic acid; 166 – linalool-1-oic acid

Table S4. Secondary metabolites in the nonpolar fraction of sea buckthorn **leaf** extract (FL); the listed compounds correspond to UHPLC-CAD peaks with area ≥1% of the total peak area.

No.	t_R [min]	$[M-H]^-$ (*m*/*z*)	$[M-H]^-$ formula	Tentative identification	Relative peak area (%)	Ref.
1	2.5	935.0785, 1103.0858	$C_{41}H_{27}O_{26}$, $C_{48}H_{31}O_{31}$	stachyurin, casuarinin hippophaenin B or isomers	1.2	
2	16.5	1459.7109	$C_{71}H_{111}O_{31}$	triterpenoid saponin	1.6	
3	16.7	1313.6537	$C_{65}H_{101}O_{27}$	triterpenoid saponin	1.8	
4	16.9	1313.6541	$C_{65}H_{101}O_{27}$	triterpenoid saponin	2.0	
5	17.7	1297.6602	$C_{65}H_{101}O_{26}$	triterpenoid saponin	1.3	
6	17.7	1401.7086	$C_{69}H_{109}O_{29}$	triterpenoid saponin	4.2	
7	17.8	1297.6603	$C_{65}H_{101}O_{26}$	triterpenoid saponin	2.0	
8	18.0	1151.6021	$C_{59}H_{91}O_{22}$	triterpenoid saponin	1.1	
9	18.4	1255.6474	$C_{63}H_{99}O_{25}$	triterpenoid saponin	4.7	
10	18.5	487.3440	$C_{30}H_{47}O_{5}$	triterpenoid	18.3	
11	19.5	1239.6549	$C_{63}H_{99}O_{24}$	triterpenoid saponin	4.3	
12	20.1	1093.5979	$C_{57}H_{89}O_{20}$	triterpenoid saponin	3.6	
13	22.4	471.3479	$C_{30}H_{47}O_{4}$	triterpenoid	2.1	[4]
14	22.4	471.3479	$C_{30}H_{47}O_{4}$	triterpenoid	3.0	[4]
15	22.6	633.3793	$C_{39}H_{53}O_{7}$	acylated triterpenoid*	2.2	[5]
16	23.0	471.3480	$C_{30}H_{47}O_{4}$	triterpenoid	13.1	[4]
17	23.2	471.3481	$C_{30}H_{47}O_{47}$	triterpenoid	7.1	[4]

* acylated with phenolic acids

Table S5. Secondary metabolites in the phenolic-rich fraction of sea buckthorn **twig** extract (GF); the listed compounds correspond to UHPLC-CAD peaks with area ≥1% of the total peak area.

No.	t_R [min]	$[M-H]^-$ (*m/z*)	$[M-H]^-$ formula	Tentative identification	Relative peak area (%)	Ref.
1	1.4	305.0659	$C_{15}H_{13}O_7$	(epi)gallocatechin	6.8	[6]
2	1.5	593.1289	$C_{30}H_{25}O_{13}$	(epi)C-(epi)GC	3.6	[7,8]
3	1.6	881.1921	$C_{45}H_{37}O_{19}$	(epi)C-(epi)C-(epi)GC	1.8	[7,8]
4	1.8	881.1922	$C_{45}H_{37}O_{20}$	(epi)C-(epi)C-(epi)GC	1.1	[7,8]
5	2.2	577.1340	$C_{30}H_{25}O_{12}$	dimeric proanthocyanidin	8.8	[7,8,9]
6	2.3	289.0709	$C_{15}H_{13}O_6$	catechin	10.0	[6,9]
7	2.7	1153.2607	$C_{60}H_{49}O_{24}$	tetrameric proanthocyanidin	2.9	[6]
8	3.1	865.1973	$C_{45}H_{37}O_{18}$	trimeric proanthocyanidin	3.6	[7,8,9]
9	3.3	577.1344	$C_{30}H_{25}O_{12}$	dimeric proanthocyanidin	2.5	[7,8,9]
10	3.6	1153.2605	$C_{60}H_{49}O_{24}$	tetrameric proanthocyanidin	1.2	[7]
11	4.6	865.1973	$C_{45}H_{37}O_{18}$	trimeric proanthocyanidin	1.4	[7,8,9]
12	4.8	300.9983	$C_{14}H_5O_8$	ellagic acid	1.2	
13	11.3	582.2600		nitrogen-containing compound	1.6	
14	11.8	612.2710		nitrogen-containing compound	2.0	
15	12.1	642.2822		nitrogen-containing compound	3.2	
16	12.5	672.2918		nitrogen-containing compound	3.9	
17	18.5	487.3430	$C_{30}H_{47}O_5$	triterpenoid	3.0	

(epi)C – (epi)catechin; (epi)GC – (epi)gallocatechin

Table S6. Secondary metabolites in the nonpolar fraction of sea buckthorn **twig** extract (GL); the listed compounds correspond to UHPLC-CAD peaks with area ≥1% of the total peak area.

No.	t_R [min]	$[M-H]^-$ (*m/z*)	$[M-H]^-$ formula	Tentative identification	Relative peak area (%)	Ref.
1	18.5	487.3437	$C_{30}H_{47}O_5$	triterpenoid	5.9	
2	19.5	487.3428	$C_{30}H_{47}O_5$	triterpenoid	1.5	
3	22.5	471.3469	$C_{30}H_{47}O_4$	triterpenoid	3.4	[4]
4	23.0	471.3464	$C_{30}H_{47}O_4$	triterpenoid	6.5	[4]
5	23.2	471.3470	$C_{30}H_{47}O_4$	triterpenoid	8.5	[4]
6	24.9	617.3839	$C_{39}H_{53}O_6$	acylated triterpenoid*	1.9	[5]
7	25.1	617.3836	$C_{39}H_{53}O_6$	acylated triterpenoid*	3.1	[5]
8	25.3	617.3842	$C_{39}H_{53}O_6$	acylated triterpenoid*	2.8	[5]
9	25.4	617.3846	$C_{39}H_{53}O_6$	acylated triterpenoid*	2.0	[5]
10	25.8	455.3527	$C_{30}H_{47}O_3$	triterpenoid	2.3	
11	26.0	617.3839	$C_{39}H_{53}O_6$	acylated triterpenoid*	11.5	[5]

* acylated with phenolic acids

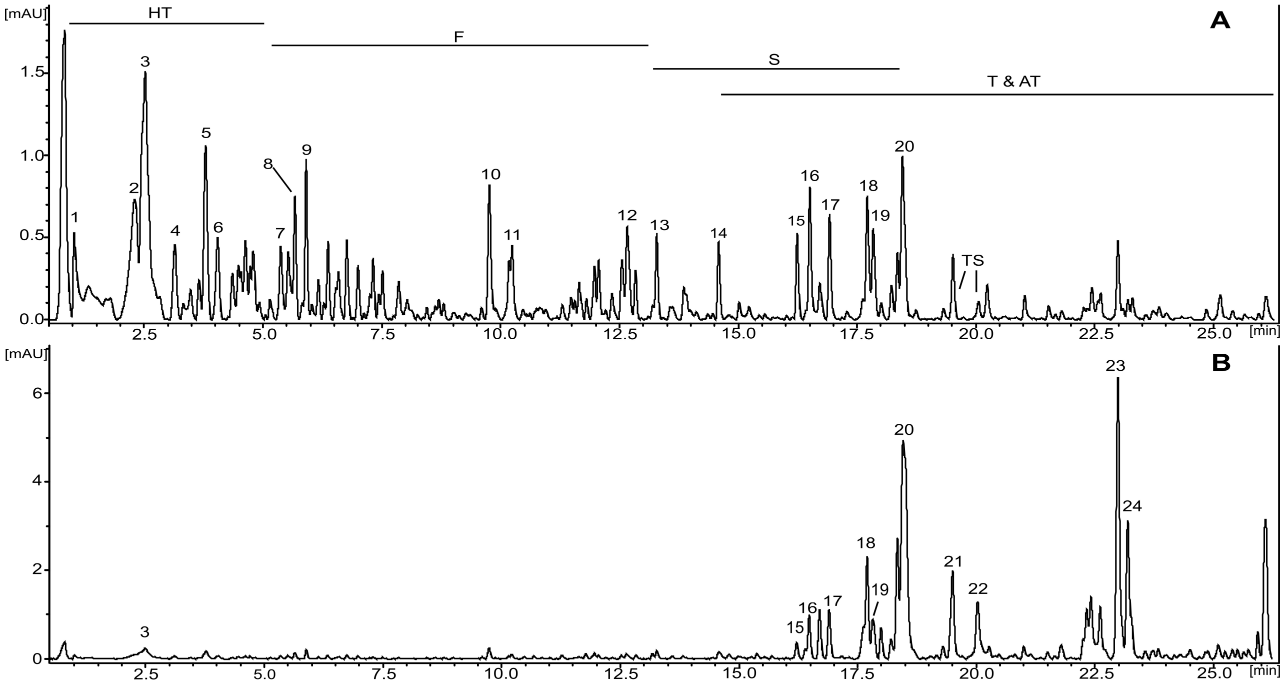

Figure S1. UHPLC-CAD chromatograms of the phenolic-rich fraction (LF) (**A**) and the nonpolar fraction (LL) (**B**) from *E. rhamnoides* (L.) A. Nelson leaves. Major peaks: 1 – GalA-Hex; 2 – strictinin / isomer; 3 – stachyurin, casuarinin, hippophaenin B / isomers; 4 – ellagitannin $C_{34}H_{26}O_{22}$; 5 – casuarictin / isomer; 6 – ellagitannin $C_{49}H_{36}O_{31}$; 7 – rutin; 8 – I-dHex-Hex; 9 – I-3-*O*-Glu-7-*O*-Rha; 10 & 11 – K-Hex-*p*CouA; 12 – I-dHex-Hex-166; 13 – S $C_{59}H_{96}O_{27}$; 14 – S $C_{65}H_{106}O_{31}$; 15 – S $C_{59}H_{96}O_{26}$; 16 – S $C_{71}H_{112}O_{31}$; 17 – S $C_{65}H_{102}O_{27; 18}$; 18 – S $C_{69}H_{110}O_{29}$; 19 – S $C_{65}H_{102}O_{26}$; 20 – T $C_{30}H_{48}O_{5}$; 21 – S $C_{63}H_{100}O_{24}$; 22 – S $C_{57}H_{90}O_{20}$; 23 & 24 – T $C_{30}H_{48}O_{4}$. HT – hydrolysable tannins; F – flavonoids; S – saponins; T – triterpenoids AT – acylated triterpenoids; I – isorhamnetin; K – kaempferol; Q – quercetin; dHex – deoxyhexose; Hex – hexose; Glc – glucose; Rha – rhamnose; GalA – gallic acid; pCouA – *p*-coumaric acid; 166 – linalool-1-oic acid.

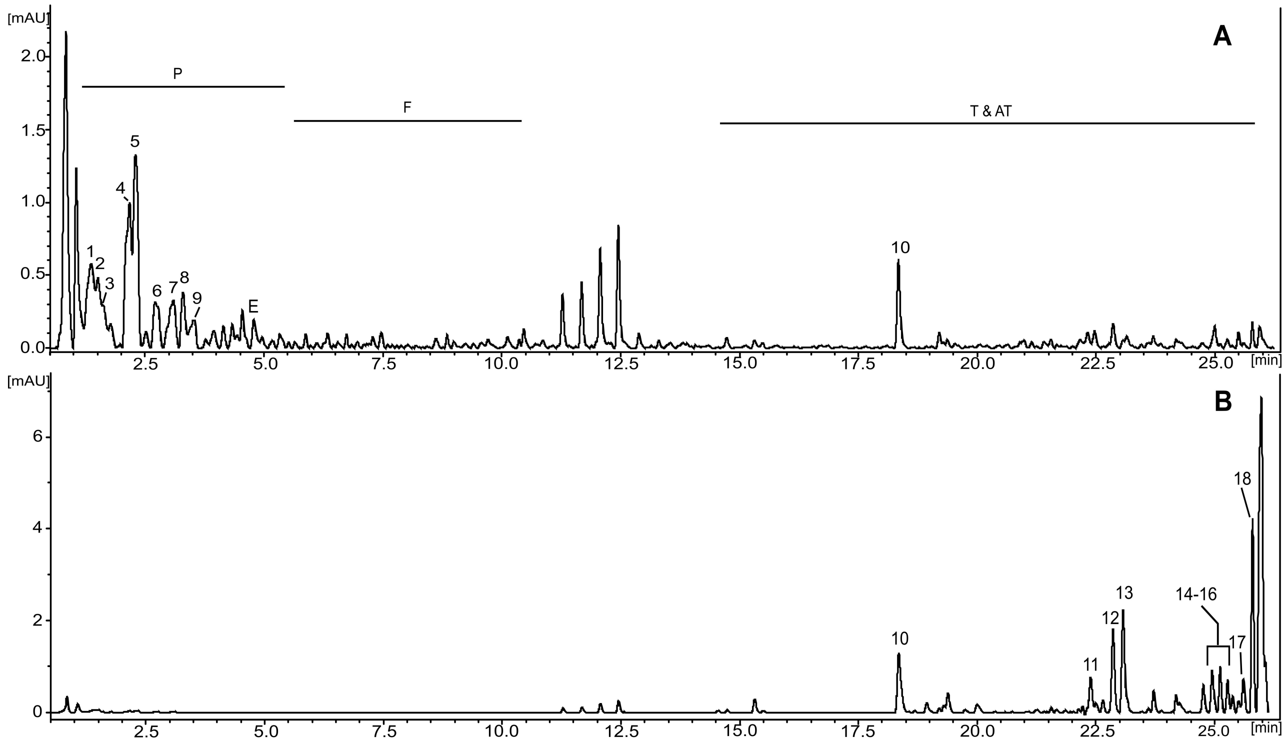

Figure S2. UHPLC-CAD chromatograms of the phenolic-rich fraction (GF) (**A**) and the nonpolar fraction (GL) (**B**) from *E. rhamnoides* (L.) A. Nelson twigs. Major peaks: 1 – (epi)GC; 2 – (epi)C-(epi)GC; 3 – (epi)C-(epi)C-(epi)GC; 4 – (epi)C-(epi)C; 5 – catechin; 6 - (epi)C-(epi)C-(epi)C-(epi)C; 7 – (epi)C-(epi)C-(epi)C; 8 – (epi)C-(epi)C; 9 – (epi)C-(epi)C-(epi)C-(epi)C; 10 – T $C_{30}H_{48}O_5$; 11 – T $C_{30}H_{48}O_4$; 12 – T $C_{30}H_{48}O_4$; 13 – T $C_{30}H_{48}O_4$; 14–16 – AT $C_{39}H_{54}O_6$; 17 – T $C_{30}H_{48}O_3$; 18 – AT $C_{39}H_{54}O_6$. P – proanthocyanidins; E – ellagic acid; F – flavonoids; T – triterpenoids; AT – acylated triterpenoids; (epi)C – (epi)catechin; (epi)GC – (epi)gallocatechin.

4. Discussion

UHPLC-MS analyses of phenolic-rich fractions from sea buckthorn leaves, twigs, and fruit demonstrated very distinct differences in composition of these fractions. The LF fraction consisted mainly of ellagitannins, flavonol glycosides, both simple and acylated, and triterpenoid saponins. Flavonoid and tannin profiles of LF are generally similar to those described in the scarce literature on phenolics of sea buckthorn leaves [2,3,4,10]. Saponins were previously purified from sea buckthorn seeds [11,12]. Although the presence of saponins in sea buckthorn leaves was previously detected using simple laboratory tests [13], it seems that our publication provides the first more detailed description of these compounds.

Simple flavonol glycosides and acylated flavonol glycosides were dominant compounds of the phenolic-rich fraction from sea buckthorn fruit (OF), constituting 67.1 % of the total peak area [1]. However, while simple flavonoids of the fruit were more or less similar to those from LF, its acylated flavonoid profile was completely different. Kaempferol hexosides acylated with *p*-coumaric acid (e.g. tiliroside) and isorhamnetin, quercetin, or kaempferol diglycosides acylated with linalool-1-oic acid, characteristic for the leaves, did not occur in the fruit [1,4]. Instead, OF contained isorhamnetin and quercetin glycosides, acylated with an untypical short-chain aliphatic acid [1].

In contrast, proanthocyanidins and catechin were dominant compounds of the GF fraction, flavonoids were present only in trace amounts, and saponins could be hardly detected. Similar flavan-

3-ols and proanthocyanidins were earlier found in sea buckthorn branches and bark [6,9] or sea buckthorn fruit [7].

Although phenolic-rich fractions from sea buckthorn fruit, leaf, and twig extracts differed significantly, the composition of the low-polarity fractions was more uniform. They shared similar profiles of triterpenoids and acylated triterpenoids, which is the most visible in the case of GL and OL [1]. Only LL was distinguished by the presence of numerous triterpenoid saponins. Acylated triterpenoids with the same molecular masses as those from LL, GL, and OL were previously isolated from the sea buckthorn bark [5]. Moreover, triterpenoids with molecular formulas of $C_{30}H_{48}O_4$, found in LL, GL, and OL were also detected in sea buckthorn leaves and fruit [4,14].

5. References

1. Olas, B.; Żuchowski J.; Lis, B.; Skalski, B.; Kontek, B.; Grabarczyk, Ł.; Stochmal, A. Comparative chemical composition, antioxidant and anticoagulant properties of phenolic fraction (a rich in non-acylated and acylated flavonoids and non-polar compounds) and non-polar fraction from *Elaeagnus rhamnoides* (L.) A. Nelson fruits. *Food Chem.* **2018**, *247*, 39-45, https://doi.org/10.1016/j.foodchem.2017.12.010
2. Moilanen, J; Sinkkonen, J; Salminen, J.P. Characterization of bioactive plant ellagitannins by chromatographic, spectroscopic and mass spectrometric methods. *Chemoecology* **2013**, *23*, 165-179, https://doi.org/10.1007/s00049-013-0132-3
3. Moilanen, J; Koskinen, P, Salminen, J.P. Distribution and content of ellagitannins in Finnish plant species. *Phytochemistry* **2015**, *116*, 188-197, https://doi.org/10.1016/j.phytochem.2015.03.002
4. Yang, Z.G.; Wen, X.F.; Li, Y.H.; Matsuzaki, K.; Kitanaka, S. Inhibitory effects of the constituents of *Hippophae rhamnoides* on 3T3-L1 cell differentiation and nitric oxide production in RAW264. 7 cells. *Chem. Pharm. Bull.* **2013**, *61*, 279-285, https://doi.org/10.1248/cpb.c12-00835
5. Yang, Z.G.; Li, H.R.; Wang, L.Y.; Li, Y.H.; Lu, S.G.; Wen, X.F.; Wang, J.; Daikonya,A.; Kitanaka, S. Triterpenoids from *Hippophae rhamnoides* L. and their nitric oxide production-inhibitory and DPPH radical-scavenging activities. *Chem. Pharm. Bull.* **2007**, *55*, 15-18, https://doi.org/10.1248/cpb.55.15
6. Yasukawa, K.; Kitanaka, S.; Kawata, K.; Goto, K. Anti-tumor promoters phenolics and triterpenoid from *Hippophae rhamnoides*. *Fitoterapia* **2009**, *80*, 164-167, https://doi.org/10.1016/j.fitote.2009.01.006
7. Kallio, H.; Yang, W.; Liu, P.; Yang, B. Proanthocyanidins in wild sea buckthorn (*Hippophae rhamnoides*) berries analyzed by reversed-phase, normal-phase, and hydrophilic interaction liquid chromatography with UV and MS detection. *J. Agric. Food Chem.* **2014**, *62*, 7721-7729, http://dx.doi.org/10.1021/jf502056f
8. Ge, Y.W.; Zhu, S.; Kazuma, K.; Wei, S.L.; Yoshimatsu, K.; Komatsu, K. Molecular ion index assisted comprehensive profiling of B-type oligomeric proanthocyanidins in rhubarb by high performance liquid chromatography-tandem mass spectrometry. *Anal. Bioanal. Chem.* **2016**; *408*, 3555-3570 https://doi.org/10.1007/s00216-016-9433-z
9. Xu, X.; Xie, B.; Pan, S.; Yang, E.; Wang, K.; Cenkowski, S.; Hydamake, A.W.; Rao, S. A new technology for extraction and purification of proanthocyanidins derived from sea buckthorn bark. *J. Sci. Food Agric.* **2006**, *86*, 486-492, https://doi.org/10.1002/jsfa.2339
10. Pop, R.M.; Socaciu, C.; Pintea, A.; Buzoianu, A.D.; Sanders, M.G.; Gruppen, H.; Vincken, J.P. UHPLC/PDA–ESI/MS analysis of the main berry and leaf flavonol glycosides from different Carpathian *Hippophae rhamnoides* L. varieties. *Phytochem. Anal.* **2013**, *24*, 484-492, https://doi.org/10.1002/pca.2460
11. Chen, C.; Gao, W.; Cheng, L.; Shao, Y.; Kong, D.Y. Four new triterpenoid glycosides from the seed residue of *Hippophae rhamnoides* subsp. *sinensis*. *J. Asian. Nat. Prod. Res.* **2014**, *16*, 231-239, https://doi.org/10.1080/10286020.2013.879383
12. Gao, W.; Chen, C.; Zhang, J. Cheng, L.; Kong D.Y. Two new triterpene saponins from the seed residue of *Hippophae rhamnoides* L. *Helv. Chim. Acta* **2015**, *98*, 60-66, https://doi.org/10.1002/hlca.201400124
13. Gupta, D.; Kaul, V. Qualitative analysis of bioactive compounds in leaves of *Hippophae rhamnoides* L. *Natl. Acad. Sci. Lett.* **2013**, *36*, 477-481, https://doi.org/10.1007/s40009-013-0160-0
14. Zheng, R.X.; Xu, X. D.; Tian, Z.; Yang, J.S. Chemical constituents from the fruits of *Hippophae rhamnoides*. *Nat. Prod. Res.* **2009**, **23**, 1451–1456, https://doi.org/10.1080/14786410903075457
